# Reengineering anthrax toxin protective antigen for improved receptor-specific protein delivery

**DOI:** 10.1186/s12915-020-00827-y

**Published:** 2020-08-13

**Authors:** Lukas Becker, Wouter P. R. Verdurmen, Andreas Plückthun

**Affiliations:** 1 Department of Biochemistry, University of Zurich, Winterthurerstr. 190, 8057 Zurich, Switzerland; 2 Department of Biochemistry, Radboud Institute for Molecular Life Sciences (RIMLS), Radboud University Medical Center, Geert Grooteplein 28, 6525 GA Nijmegen, The Netherlands

**Keywords:** Anthrax toxin, Protective antigen, Cytosolic protein delivery, DARPin

## Abstract

**Background:**

To increase the size of the druggable proteome, it would be highly desirable to devise efficient methods to translocate designed binding proteins to the cytosol, as they could specifically target flat and hydrophobic protein-protein interfaces. If this could be done in a manner dependent on a cell surface receptor, two layers of specificity would be obtained: one for the cell type and the other for the cytosolic target. Bacterial protein toxins have naturally evolved such systems. Anthrax toxin consists of a pore-forming translocation unit (protective antigen (PA)) and a separate protein payload. When engineering PA to ablate binding to its own receptor and instead binding to a receptor of choice, by fusing a designed ankyrin repeat protein (DARPin), uptake in new cell types can be achieved.

**Results:**

Prepore-to-pore conversion of redirected PA already occurs at the cell surface, limiting the amount of PA that can be administered and thus limiting the amount of delivered payload. We hypothesized that the reason is a lack of a stabilizing interaction with wild-type PA receptor. We have now reengineered PA to incorporate the binding domain of the anthrax receptor CMG2, followed by a DARPin, binding to the receptor of choice. This construct is indeed stabilized, undergoes prepore-to-pore conversion only in late endosomes, can be administered to much higher concentrations without showing toxicity, and consequently delivers much higher amounts of payload to the cytosol.

**Conclusion:**

We believe that this reengineered system is an important step forward to addressing efficient cell-specific delivery of proteins to the cytosol.

## Background

Targeted therapy is nowadays employed to treat several kinds of diseases in which aberrant signaling plays an important role. The molecular targets are typically of two types. The first group are cell surface molecules that are targeted with antibodies, which display a variety of mechanisms of action, including the inhibition of signaling, recruitment of immune functions, or of other molecules, or they can be coupled to toxins and form antibody-drug conjugates (ADCs). The second group of drug targets is intracellular, exemplified by kinases, which are targeted by small molecules that are inherently cell-permeable, and bind to small pockets on their target protein. While all of these approaches have shown great promise, lack of a sufficient therapeutic window and rapid development of resistance are common problems [1–4].

In contrast to extracellular targets that are well accessible to antibodies or other binding proteins, intracellular protein-protein interactions represent a largely untapped resource of targets for cell-specific targeted therapy [1, 2]. Small molecules can be developed with high specificity and affinity for many intracellular proteins that provide pockets, a success of decades of development of medicinal chemistry. However, small molecules can usually not inhibit protein-protein interactions, since they cannot bind with high enough specificity to hydrophobic and flat protein-protein interfaces that lack deep binding pockets [2]. Furthermore, small molecules can be target-specific, but not cell-specific.

Binding proteins can be generated today against basically any target molecule, but as therapeutics are mostly limited to targets accessible on the cell surface due to the impermeability of the plasma membrane to biological macromolecules, including proteins. Various delivery methods based on naturally occurring systems as well as on non-natural systems are being developed to deliver proteins across the plasma membrane, yet with widely varying effectiveness, thus aiming to increase the druggable proteome [3].

Bacterial protein toxins, e.g., anthrax toxin (from *Bacillus anthracis*), have evolved naturally to overcome this barrier, the plasma membrane, and are able to transport protein toxins to the cytosol of cells in a receptor-specific manner. Upon receptor binding and proteolytic activation in anthrax toxin-mediated delivery, the toxin complex gets internalized via receptor-mediated endocytosis. In the endosomes, cargo molecules get translocated directly to the cytosol or to intraluminal vesicles and they eventually reach the cytosol by back fusion of these vesicles [4–7]. Due to the modular structure of these toxins, domains can be engineered for altered cell specificity and translocated cargo, making it an adaptable system for protein delivery [8–12]. 

Recently, our group has developed a generic delivery system based on anthrax toxin, able to deliver a set of model binding proteins to the cytosol of cells [10]. For retargeting the cell-binding and translocation domain of anthrax toxin, a designed ankyrin repeat protein (DARPin) which binds to the epithelial cell adhesion molecule (EpCAM) was fused C-terminally to protective antigen (PA) with a receptor binding-ablated domain 4 (carrying mutations N682A, D683A), termed PA_m_, for mutated PA. The PA-binding domain of one of the two native anthrax toxin cargoes, lethal factor 1-254 (LF_N_), was fused N-terminally to different cargo DARPins. With this retargeting strategy, we successfully delivered these cargo DARPins to the cytosol of EpCAM-expressing cells [10].

For retargeted PA, however, only low concentrations could be used, due to a cytotoxic effect of PA alone that occurred with higher concentrations (> 20 nM). Our aim was therefore to generate an in-depth understanding of the underlying mechanism of this toxicity and use this knowledge to design novel reengineered protective antigen variants that overcome the cytotoxic limitations of retargeting and thus to be able to deliver higher quantities of payload. Inspired by the notion that the interface between domains 2 and 4 in the wild-type PA prepore is stabilized by binding to its natural receptor [13], we rationalized that the cytotoxicity is most likely due to a premature prepore-to-pore conversion of PA, already at physiological pH [13, 14]. To counter this effect, we now generated a stabilized version of PA, which contains PA in its wild-type form (PA_wt_) with the wild-type soluble extracellular receptor-binding domain of PA, fused to a retargeting DARPin.

Here, we provide a detailed protein characterization, confirm the elimination of the cytotoxicity, and show a higher uptake of cytosolically delivered proteins with the new fusion construct. We show that the amount of cytosolically delivered cargo was so far limited by the cytotoxicity of the translocation domain and that this rate-limiting step has now been overcome.

## Results

### Design of PA_wt_-sANTXR-Ac2

**Fig. 1:**
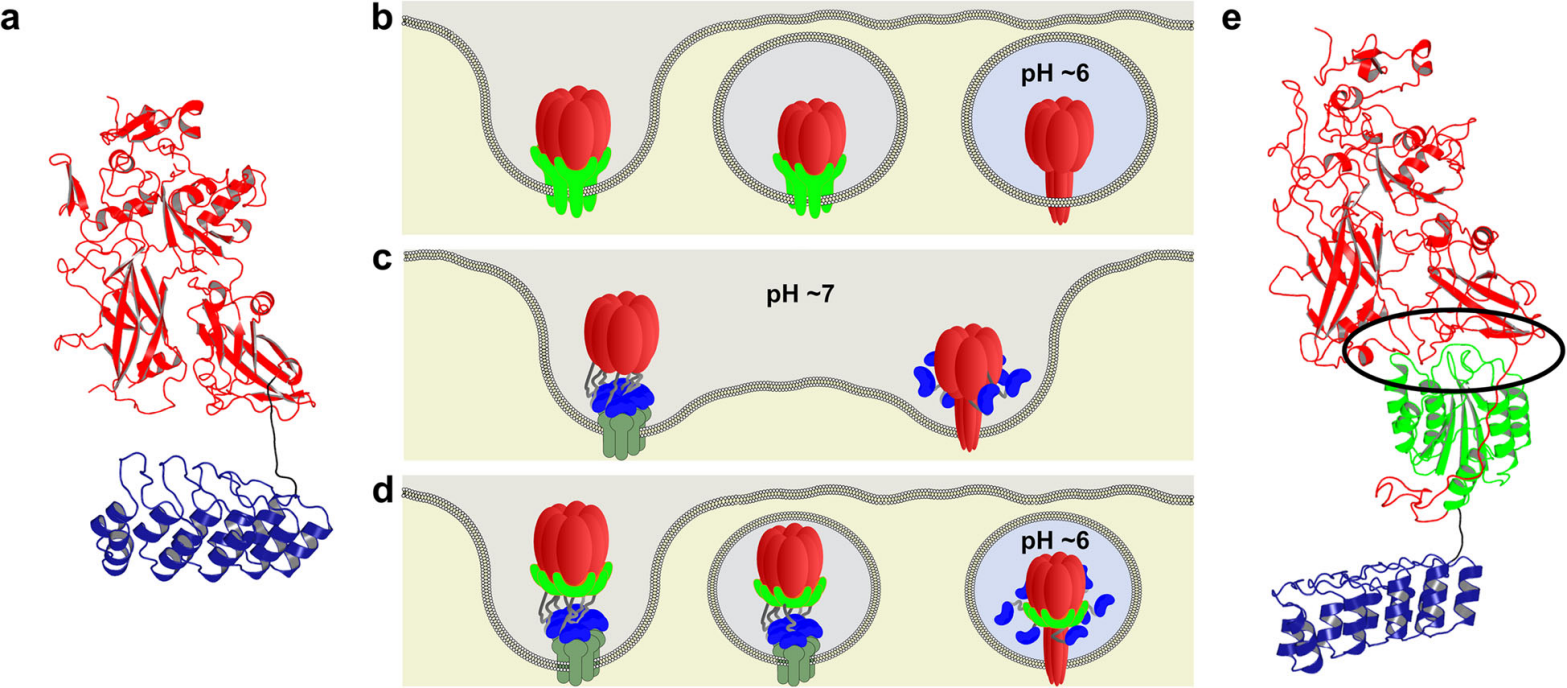
Ribbon representation of the structures of PA constructs shown in their activated/furin-cleaved PA_63_ version. **a** Previously published, retargeted PA_m_-Ac2 [10]. **b**–**d** Schematic representation of the prepore-to-pore conversion at the respective pH of furin-cleaved PA_wt_ (**b**), PA_m_ fused to a retargeting DARPin, PA_m_-Ac2 (**c**), and PA_wt_ fused to the wild-type receptor domain and the retargeting DARPin, PA_wt_-sANTXR-Ac2 (**d**). **e** Newly designed stabilized PA_wt_-sANTXR-Ac2 with PA_wt_, the wild-type receptor CMG2 VWA domain, and the retargeting DARPin; PA shown in red, EpCAM-retargeting DARPin Ac2 shown in blue, CMG2 receptor VWA domain (sANTXR) shown in green, and prepore-stabilizing interaction region highlighted in black oval. Protein structures were adapted from PDB ID: 1TZN (PA prepore binding sANTXR), 1ACC (PA), and 4YDW (DARPin)

Retargeting of PA to various cell surface receptors has previously been achieved by fusing a binding protein to the C-terminus of PA, and we have developed such strategy using DARPins [10]. Having fused an EpCAM-targeting DARPin (Ac2) with an affinity of 1.3 × 10^−7^ M [15] to the C-terminus of a mutated version of PA, ablating binding to its own receptors, capillary morphogenesis gene-2 (CMG2) and tumor endothelial marker-8 (TEM8) (Fig. 1a), we generated a highly efficient, cell-specific, retargeted delivery system. Even with low concentrations (20 nM) of the retargeting fusion construct PA_m_-Ac2, we could detect the cytosolic presence of cargo DARPins [10]. When increasing the concentration of PA_m_-Ac2, however, we observed that our delivery system was highly toxic for Flp-In 293-EpCAM-BirA cells stably overexpressing the targeted receptor, without any toxic cargo being delivered. Therefore, we performed an in-depth analysis of PA_m_-Ac2 to search for the possible cytotoxic mechanism and measures to overcome this.

When domain 4 of PA_wt_ binds to the wild-type receptor, it forms a metal-ion-dependent structural bridge between domain 4 and the von Willebrand factor A (VWA) region of the anthrax toxin receptor (CMG2 or TEM8) (Fig. 1b). Especially two binding residues (N682, D683) within domain 4 are very important for PA binding [16]. Although receptor binding is mainly mediated by domain 4 of PA, parts of the VWA region also interact with domain 2. Binding to the 340-348 loop of PA prevents the rearrangement of the PA insertion loop and the contiguous 2β2 and 2β3 β-strands.

It has been shown that the prepore-to pore conversion of PA_wt_ occurs at different pH, depending on it being incubated with or without its wild-type receptor [13, 17]. Using mutated PA (PA_m_), which is unable to bind its wild-type receptor, the stabilizing interactions between domain 2 and the VWA region are lost, which otherwise prevent the conformational change at neutral pH. Thus, merely fusing a retargeting molecule to PA_m_ does not fully replicate the mechanism of PA_wt_, which limits the conformational changes to occur in late endosomes. Hence, we propose that the prepore-to-pore conversion of PA_m_-Ac2 can occur immediately upon oligomerization on the cellular surface, already at physiological pH, thus assembling an open pore allowing ions and other substances to freely pass in and out of the cell (Fig. 1c).

To prevent this premature prepore-to-pore conversion, we designed a domain-2/domain-4 interface-stabilized version of PA (Fig. 1d, e). To achieve this, we genetically fused the 19.5-kDa VWA domain of CMG2 (residues 40-217, C175A), which we termed sANTXR, to the C-terminus of PA_wt_. A long (G_4_S)_5_ linker between PA_wt_ and sANTXR with an approximate length of 88 Å allows the correct orientation and functional interaction of the fusion partners. The covalent linker massively increases the local effective concentration of sANTXR, which in combination with the high affinity for the PA-binding domain is expected to effectively reduce off-target effects of PA_wt_ binding to CMG2 or TEM8 on the cell surface [18]. This was deduced from the structure of the wild-type conformation of the PA prepore [13], PDB ID: 1TZN. C-terminally to the sANTXR receptor domain, we fused the EpCAM-targeting DARPin Ac2. We propose that the sANTXR domain impedes premature prepore-to-pore conversion by creating a very similar domain arrangement as in PA_wt_ bound to its receptor CMG2. We thus expect that the pH where the prepore-to-pore conversion can occur shifts back to wild-type conditions (Fig. 1b–d), conditions that are present only in the (late) endosomes. The cytotoxicity of a premature prepore-to-pore conversion on the cell surface thus should get diminished.

To confirm that the stabilizing interaction is really due to the functional interaction of PA with the wild-type receptor domain, we designed a PA mutant construct, PA_m_-sANTXR-Ac2, with the mutations N682A and D683A (Additional file 1: Figure S1), which should prevent binding of PA_m_ and sANTXR, thus having no stabilizing interaction. As another control, we also designed a variant with a very short linker between PA_wt_ and the sANTXR domain, restraining the sANTXR domain to an orientation in which binding of PA_wt_ to sANTXR is sterically prevented. Comparing these constructs, a functional dependency of the stabilizing interaction and prepore-to-pore conversion was tested.

### PA_wt_-sANTXR-Ac2 reduces cytotoxicity and is dependent on functional interaction of PA_wt_ with its wild-type receptor domain

**Fig. 2:**
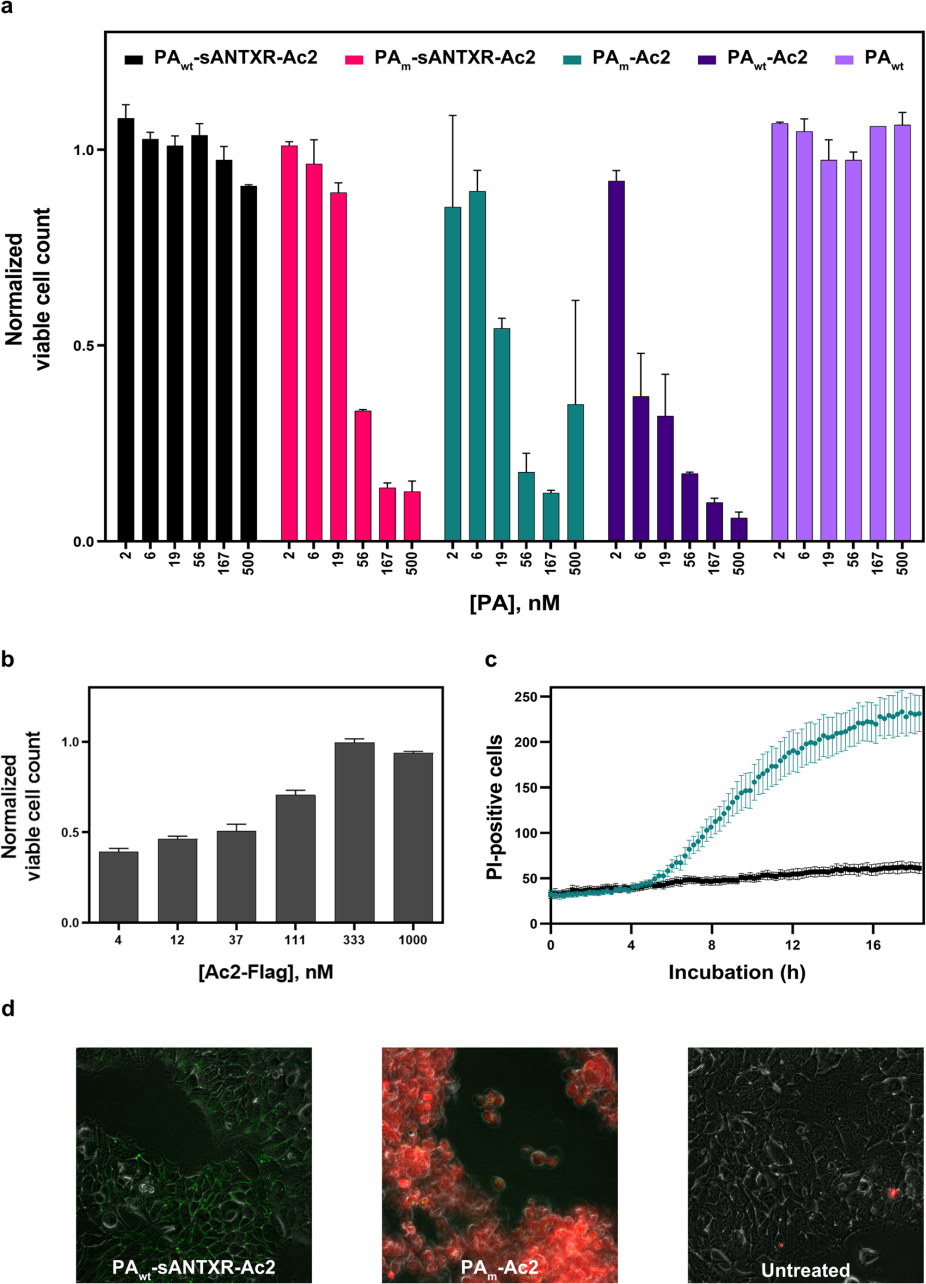
Cytotoxicity of different PA variants in comparison with PA_wt_-sANTXR-Ac2. **a** Viability assay of Flp-In 293-EpCAM-BirA with respective concentrations of PA_wt_-sANTXR-Ac2, PA_m_-sANTXR-Ac2, PA_m_-Ac2, PA_wt_-Ac2, and PA_wt_ (*n* = 3). Error bars indicate SEM. **b** Competition assay with Ac2 DARPin and 100 nM PA_m_-Ac2 with increasing amounts of competitor Ac2-Flag (*n* = 3). Error bars indicate SEM. **c** Quantification of propidium iodide (PI)-positive Flp-In 293-EpCAM-BirA cells during time-lapse imaging, cells treated with either PA_wt_-sANTXR-Ac2 (black squares) or PA_m_-Ac2 (green circles) (*n* = 6). Error bars indicate SEM. **d** PI (red) staining for permeable cells and LF_N_-eGFP staining (green) for binding to surface PA, comparing PA_wt_-sANTXR-Ac2 and PA_m_-Ac2 on Flp-In 293-EpCAM-BirA cells

We tested the cytotoxicity of our previously developed construct, PA_m_-Ac2, in comparison to the new construct PA_wt_-sANTXR-Ac2. Upon incubation of Flp-In 293-EpCAM-BirA cells, which have been made to stably overexpress EpCAM, with increasing concentrations of PA_wt_-sANTXR-Ac2, no change in cellular viability was observed up to 500 nM, the maximal concentration tested (Fig. 2a). Flp-In 293-EpCAM-BirA cells, when incubated with PA_m_-Ac2 (not containing the receptor domain fusion), however, showed a decrease in viability of ~ 50% already at 19 nM, and even down to only 10% viability at a concentration of 167 nM PA_m_-Ac2. PA_m_-sANTXR-Ac2 (which comprises the mutated, non-interacting domains) showed a similar reduction of viability for concentrations ≥ 56 nM, confirming the necessity of a functional interaction between the VWA domain and PA_wt_. PA_m_-sANTXR-Ac2 appears to be less toxic than PA_m_-Ac2, presumably due to steric hindrance of the slightly larger fusion construct, impeding pore formation. We observed in time-lapse imaging video microscopy that the cytotoxicity with this construct occurs at a later timepoint, as described below (Additional file 1: Figure S2, showing the analysis of the videos of Additional files 2 and 3). To ensure that the toxicity was not due to the mutations associated with PA_m_, we used PA_wt_-Ac2 as a further control, which, in addition to binding CMG2, will bind EpCAM via Ac2. We expected a comparable toxicity of PA_wt_-Ac2 and PA_m_-Ac2 on Flp-In 293-EpCAM-BirA, since binding will be mostly via the highly overexpressed EpCAM without prepore stabilization, and only to a limited extent via CMG2 and TEM8. Indeed, PA_wt_-Ac2 shows a similar toxicity as PA_m_-Ac2. A non-targeted control, without the EpCAM binding DARPin Ac2, had no effect on the cells.

To confirm the receptor-specific cytotoxicity of the PA prepore, we incubated Flp-In 293-EpCAM-BirA cells with 100 nM PA_m_-Ac2, which showed clear toxic effects (Fig. 2a), and titrated the DARPin Ac2 (Ac2-FLAG) as a binding competitor. With increasing concentrations of competitor, the cytotoxicity was reduced, and with a ~ 3-fold excess of Ac2 DARPin over PA_m_-Ac2, 100% viability was restored, indicating the cytotoxicity is due to the interaction of PA_m_-Ac2 with EpCAM and not due to a non-specific cytotoxic effect (Fig. 2b).

In addition to the cell proliferation assay, we performed time-lapse imaging over 18 h. Flp-In 293-EpCAM-BirA cells were treated with 100 nM PA_wt_-sANTXR-Ac2 or PA_m_-Ac2, propidium iodide (PI), a marker of cell death, and eGFP fused to the C-terminus of LF_N_, LF_N_-eGFP. Cells were imaged over time with an automated LionHeart FX microscope. We measured the increase in PI staining for PA_wt_-sANTXR-Ac2 and PA_m_-Ac2 (Fig. 2c and Additional files 4 and 5). Up to 250 cells are PI positive in wells incubated with PA_m_-Ac2 in a time-dependent manner, while PA_wt_-sANTXR-Ac2 remained constant at the initial number of ~ 50 PI-positive cells. The lag in response time immediately after addition of PA variants can be attributed to the binding and pore formation on the cell surface, as well as the tolerance of cells to a certain number of pores formed on the plasma membrane (Additional file 1: Figure S3). We also confirmed cell death by PI staining with confocal microscopy. Cells were treated with 100 nM of the respective constructs and incubated for 3 h before confocal imaging. PA_wt_-sANTXR-Ac2 shows no cytotoxicity and is thus indistinguishable from untreated control cells, while cells treated with PA_m_-Ac2 detach and stain highly positive for PI (Fig. 2d).

With the control PA_m_-sANTXR-Ac2 (without functional interface between these components), we observed a slight delay in cytotoxicity in initial time-lapse imaging compared to PA_m_-Ac2 (Additional file 1: Figure S2). We propose that the slightly larger receptor fusion construct PA_m_-sANTXR-Ac2 sterically hinders rapid prepore-to-pore conversion on the cell surface.

To further investigate the structure-function relationship, we designed a construct with a very short linker (SL) between PA_wt_ and the wild-type receptor domain, preventing the correct orientation and binding of PA_wt_ to the VWA domain. With this construct, PA_wt-SL_-sANTXR-Ac2 (Additional file 1: Figure S1), we performed a viability assay and could observe a reduced cell viability to 63% at 580 nM (Additional file 1: Figure S4a). The higher concentrations where a cytotoxic effect is observed compared to PA_m_-Ac2 could have a similar cause as PA_m_-sANTXR-Ac2: steric hindrance with respect to form functional intramolecular complexes. To test this hypothesis, we performed a delivery assay to see if it would be still capable of prepore assembly, prepore-to-pore conversion, and delivery (see next section) as discussed below. Even though PA_wt-SL_-sANTXR-Ac2 was provided as a fusion with N-terminal His_6_-MBP, we want to point out that His_6_-MBP will be cleaved off by furin and the fusion construct, His_6_-MBP-PA_wt-SL_-sANTXR-Ac2, has previously been shown to demonstrate equivalent delivery to PA_m_-Ac2 [19].

### PA_wt_-sANTXR-Ac2 reduces cytotoxicity in a receptor expression level-dependent manner

In order to understand to what extent the cytotoxic effects of premature prepore-to-pore conversion is a function of the receptor expression level, we tested our constructs on a panel of EpCAM-positive cells, having different levels of receptor expression: HT29, MCF7, SKBR3, with EpCAM-negative RD cells as control.

**Fig. 3:**
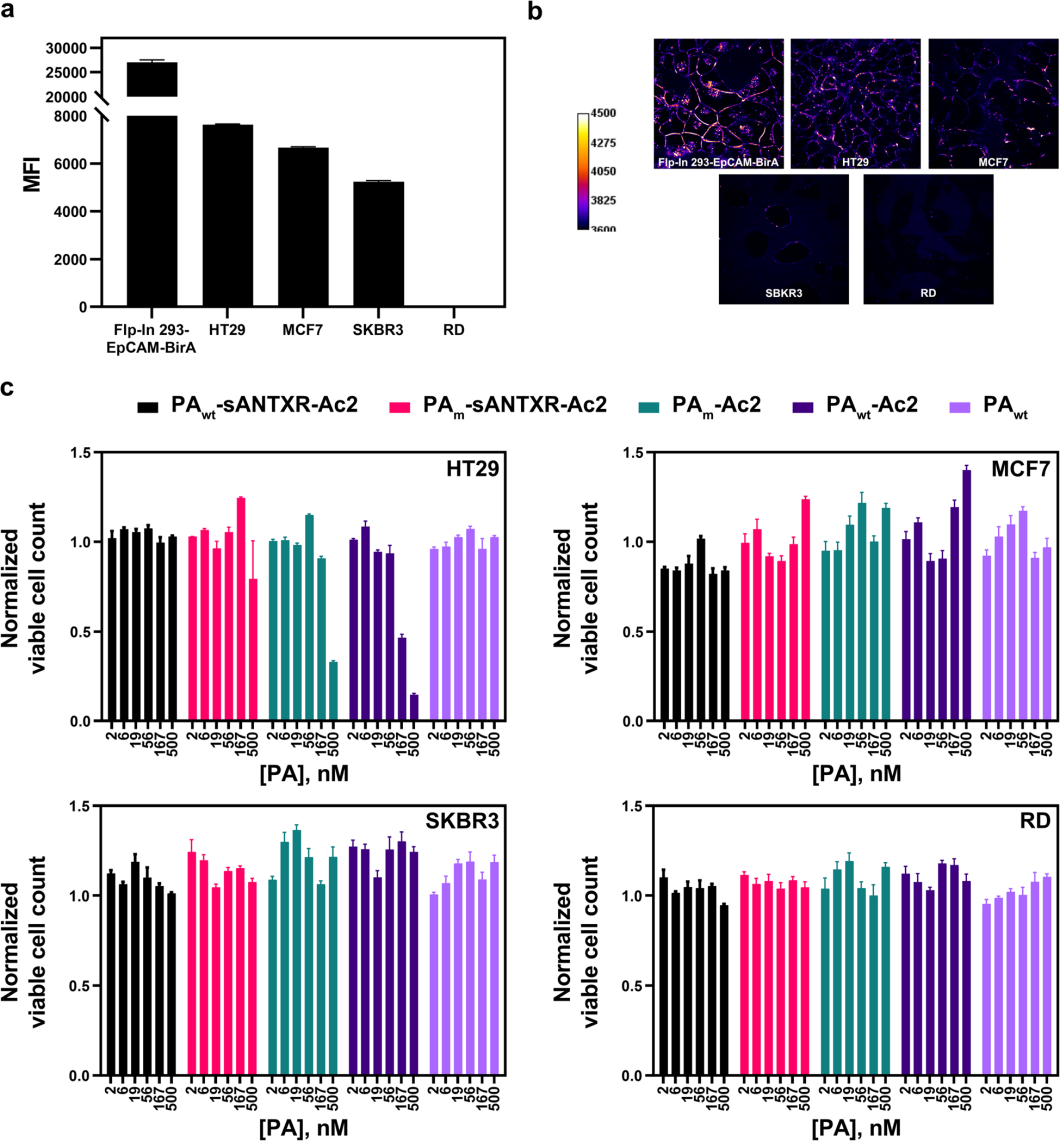
Effects of PA on different cell lines expressing EpCAM. **a** EpCAM surface expression data assessed via flow cytometry using an Alexa Fluor 488-labeled anti-EpCAM mouse mAb (*n* = 3). Error bars reflect SEM. **b** Confocal imaging of stained Flp-In 293-EpCAM-BirA cells with PA_wt_-sANTXR-Ac2 and LF_N_-eGFP to assess PA oligomerization. **c** Viability assays for a set of cell lines with PA_wt_-sANTXR-Ac2, PA_m_-sANTXR-Ac2, PA_m_-Ac2, PA_wt_-Ac2, and PA_wt_ (*n* = 3). Error bars reflect SEM

First, we assessed the EpCAM expression levels via flow cytometry using an Alexa Fluor 488-labeled anti-EpCAM mouse mAb (Fig. 3a, Additional file 1: Figure S5). EpCAM has the highest expression levels in the constructed Flp-In 293-EpCAM-BirA cells stably expressing EpCAM, followed by HT29, MCF7, SKBR3, and the EpCAM-negative RD cell line with no detectable surface EpCAM. Since Chernyavska et al. [20] recently estimated EpCAM levels of MCF7 cells at about 5.3 × 10^5^ receptors/cell, we can assume that levels of the high-expressing Flp-In 293-EpCAM-BirA cells are around 2 million receptors/cell, even though these numbers have considerable uncertainty.

We then assessed whether the receptor expression level correlates with the oligomerization and prepore formation of PA_wt_-sANTXR-Ac2. It is possible to visualize PA oligomers by saturating available binding sites with LF_N_-eGFP, which is not transported (Additional file 1: Figure S6). Using confocal microscopy, we found that a higher receptor density resulted in more prepore formation, reflecting successful PA oligomerization (Fig. 3b). The signal was highest for Flp-In 293-EpCAM-BirA cells, followed by HT29 cells. For MCF7 and especially SKBR3, however, very little signal can be detected, although the receptor expression levels are in similar ranges as for the HT29 cell line. No signal for RD cells was observed, the EpCAM-negative control cell line. For cells expressing EpCAM, we detected a membrane-like staining pattern when incubated with LF_N_-eGFP and PA_wt_-sANTXR-Ac2. For Flp-In 293-EpCAM-BirA cells and HT29 cells, we further detected a dotted staining within cellular compartments, showing endo-/lysosomal localization. Endosomal entrapment of LF_N_-eGFP has been confirmed with the BirA assay (Additional file 1: Figure S6). The detection of an endosomal-like staining for LF_N_-eGFP in MCF7 and SKBR3 cells is not evident due to the detection threshold of the microscope in combination with the limited numbers of receptors.

We propose that the non-linear dependency of PA prepore formation on receptor density is due to a receptor-level threshold below which pore formation becomes less efficient. Additionally, varying mobilities of the receptors or different internalization and degradation rates of EpCAM in the different cell lines as well as different efficiency of furin activation may also contribute to these differences [21].

We then performed a viability assay with the panel of cell lines with PA_wt_-sANTXR-Ac2, PA_m_-sANTXR-Ac2, PA_m_-Ac2, PA_wt_-Ac2, and the non-targeted control, PA_wt_ (Fig. 3c). A reduced cell viability can be observed for HT29 cells (Fig. 3c) with concentrations of 167 nM of PA_wt_-Ac2 and 500 nM of PA_m_-Ac2, leading to a viability of 46% and 33%, respectively. For MCF7, SKBR3, and RD cells, no cytotoxicity could be observed, which is in agreement with the lower expression levels of the receptor and it correlates to the expected lower levels of prepore formation on these cells.

### Lower toxicity of PA_wt_-sANTXR-Ac2 enables greater cytosolic protein delivery

Previously, we have shown that PA_m_-Ac2 can efficiently deliver various cargoes to the cytosol of Flp-In 293-EpCAM-BirA cells stably overexpressing EpCAM [10]. Our goal in this study was to increase the amount of cytosolically delivered cargo molecules, which previously was not possible, since concentrations higher than 20 nM of the pore-forming PA_m_-Ac2 drastically reduced cellular viability even within the short 4-h incubation time (Additional file 2). Our newly designed, prepore-stabilizing PA_wt_-sANTXR-Ac2 was therefore next tested for efficient protein delivery with the biotin ligase assay [22].

We incubated Flp-In 293-EpCAM-BirA cells with PA_wt_-sANTXR-Ac2, PA_m_-sANTXR-Ac2, and PA_m_-Ac2 for 4 h in the presence of the proteasome inhibitor MG-132. MG-132 was included to assess the delivery systems independently of proteasomal degradation. As cargo proteins, we tested three different DARPins, varying in size and thermostability, which have previously shown to be effectively translocated [10]. These cargo molecules contain the biotin-acceptor avi-tag and an HA-tag at their C-terminus and are fused with their N-terminus to LF_N_. Cytosolically localized cargo proteins are biotinylated by a cytoplasmically encoded BirA of Flp-In 293-EpCAM-BirA cells [22]. Cargo molecules which are trapped within the endosome, not reaching the cytosol, are not biotinylated. The HA-tag is used to determine total cellular uptake, located in the cytosol and in any other cellular compartment, allowing the determination of the intracellular localization of a cargo molecule. After cell harvest and western blotting, biotinylated cargoes were detected with streptavidin IRDye 680LT and total cellular uptake was measured via an HA-tag antibody [22]. For quantification of cytosolically present cargo molecules, the protein(s) detected at around 70 kDa, which we hypothesized earlier to be endogenous heat shock protein 70 (HSP70), were chosen as a loading control [10].

**Fig. 4:**
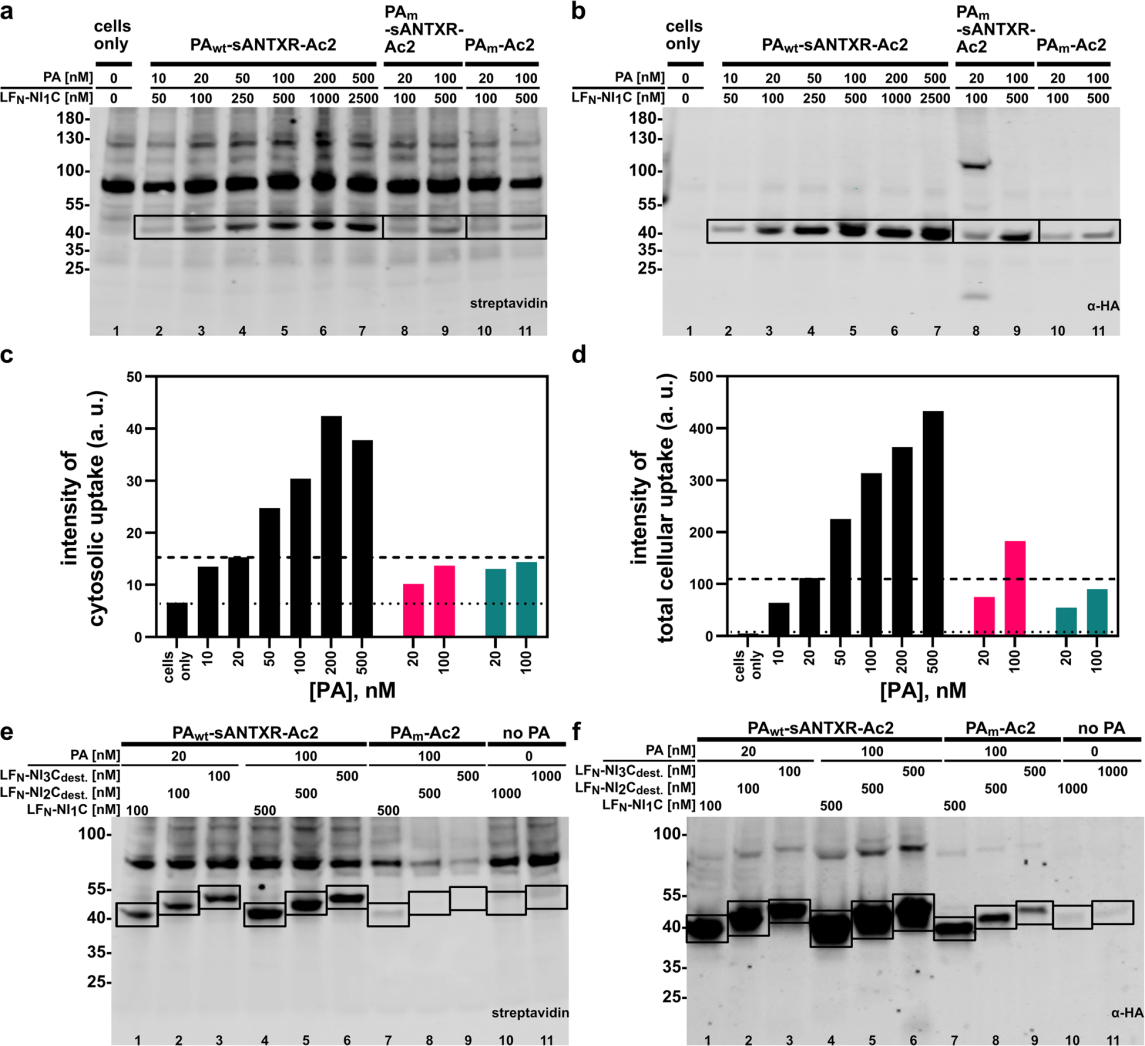
Western blots of the BirA assay showing increased delivery of LF_N_-cargo constructs with PA_wt_-sANTXR-Ac2 on Flp-In 293-EpCAM-BirA cells. Cytosolically delivered cargo proteins are biotinylated by a cytoplasmically encoded BirA and stained with Streptavidin IRDye 680LT. Total cellular uptake measured via HA-tag on the LF_N_-cargo. **a**, **b** Increasing concentrations of respective PA constructs incubated with a 5-fold excess of LF_N_-NI_1_C. Boxes indicate the bands of interest. **c**, **d** Quantification of western blot bands from **a** and **b**. Black bars, PA_wt_-sANTXR-Ac2; red bars, PA_m_-sANTXR-Ac2; green bars, PA_m_-Ac2. The dotted line represents background signal (i.e., cells only), and the dashed line shows the signal of cargo at 20 nM PA_wt_-sANTXR-Ac2. **e**, **f** Cytosolic localization (**e**) and total cellular uptake (**f**) of three different cargo DARPins delivered with 20 nM (lanes 1–3) and 100 nM (lanes 4–6) of PA_wt_-sANTXR-Ac2 or 100 nM (lanes 7–9) for PA_m_-Ac2; lanes 10 and 11 represent cells incubated with 100 nM LF_N_-cargo without PA. "dest." refers to rationally destabilized versions of NI_2_C and NI_3_C DARPins [10]. Boxes indicate the bands of interest

With increasing concentrations of PA_wt_-sANTXR-Ac2, total cellular uptake (Fig. 4b, d) and cytosolic delivery (Fig. 4a, c) of the smallest DARPin NI_1_C increase. An increase in cytosolically present cargo can be seen up to an external concentration of 200 nM. Further increases in the concentration of PA_wt_-sANTXR-Ac2 did not yield higher amounts of delivered DARPin (Fig. 4c, d), presumably due to a saturation of the receptors exploited for delivery.

At 20 nM, similar delivery efficiencies can be observed for PA_wt_-sANTXR-Ac2, PA_m_-sANTXR-Ac2, and PA_m_-Ac2, but an increase to 100 nM does not lead to an increase in cytosolically present cargo for PA_m_-sANTXR-Ac2 and PA_m_-Ac2, as it does for PA_wt_-sANTXR-Ac2, likely due to the premature prepore-to-pore conversion of PA_m_-Ac2 and PA_m_-sANTXR-Ac2 on the cell surface. This lack of functional pores renders the cells unable to unfold and translocate LF_N_-cargo proteins (Fig. 4c, d). Slightly higher total cellular uptake of LF_N_-cargo can be observed with PA_m_-sANTXR-Ac2 than for PA_m_-Ac2, probably due to the delayed cytotoxicity compared to PA_m_-Ac2.

Similar results have been observed for LF_N_-NI_2_C_dest._ as well as LF_N_-NI_3_C_dest._, constructs that have been slightly destabilized to facilitate their unfolding and refolding during transport through the pore [10, 19] (Fig. 4e, f). With increasing concentrations (20 nM and 100 nM), an increase in cytosolic cargo delivery can be observed.

The BirA assay for His_6_-MBP-PA_wt-SL_-sANTXR-Ac2 (Additional file 1: Figure S4b) showed a reduced amount of total cellular uptake, suggesting a steric inhibition effect already at the start of the internalization process. The results for this construct are in line with the results for PA_m_-sANTXR-Ac2 and confirm the functional dependency of PA on interactions with the sANTXR domain.

## Discussion

The druggable proteome is so far limited by requiring binding sites for small molecules. Macromolecular binding molecules, which do not have this restriction, are currently excluded by the lack of efficient, cell-specific cytosolic delivery systems. A solution to this problem would open up the intracellular target space for larger biological macromolecules, which can be created to almost any surface on the target. Since recombinant binding proteins are easily accessible today, a solution would drastically increase opportunities for targeted therapy approaches. Many molecules of great medical interest that are currently believed to be “undruggable targets,” since they do not have a binding pocket for small molecules, could then be targeted. Large, flat, and hydrophobic protein-protein interaction surfaces would thus remain no longer undruggable.

Previous studies have shown the utility of bacterial protein toxins as easily adaptable delivery systems. Such systems ultimately have two layers of specificity, the surface marker and the target in the cytosol, and may thus pave the way also to more specific treatments. In order to adapt bacterial protein toxins for cytosolic delivery, a thorough understanding of the wild-type delivery mechanism is absolutely necessary. The wild-type delivery mechanism of anthrax toxin has been studied in considerable detail [5]. Retargeting of anthrax toxin has been achieved by fusing different binding proteins to the C-terminus of a mutant version of PA (N682A, D683A), the anthrax toxin binding and internalization subunit, rendering it unable to bind its wild-type receptor [16]. However, the impact of this change in receptor specificity on the succeeding steps of the delivery process had not been studied.

In this study, we have conducted an in-depth analysis of a DARPin-retargeted PA, which showed a clear cytotoxicity on targeted cells when high concentrations are matched with high receptor expression levels. We therefore rationally designed an improved PA variant. This new protein design allowed us to diminish the cytotoxicity, and it highlights the importance of the interaction between PA and its wild-type receptor in controlling the conformational changes during the internalization process, tightly linking it to the pH of the internal compartments. We deduced that the interaction of PA with sANTXR, now encoded in our improved PA variant itself and no more part of the actual interaction of PA_wt_ with the surface VWA domain of CMG2 or TEM8, shifts the pH of the prepore-to-pore conversion to the wild-type conditions. The importance of this pH-sensing mechanism has been described before [13], but this knowledge had not been used in improved constructs.

Furthermore, we showed that the mechanistic concept of cytotoxicity is valid and can be rescued across multiple EpCAM-expressing cell lines, and we confirmed that cytotoxicity is also dependent on the expression level of the targeted receptor. It remains still unclear, however, why there appears to be a threshold, above which PA_m_-Ac2 shows its toxic effect. However, there are multiple factors that may explain the differences across cell lines, including different furin activity on the cell surface or differences in receptor mobility, both involved in initiating oligomerization, and there may be others, some of which have already been discussed previously [19].

It has also been shown previously that retargeting of PA to HER2 could be achieved; however, the readout in this study was based on the cytotoxicity of the cargo component [11]. Our study clearly shows that cytotoxicity might arise from the delivery system itself even if no toxic cargo is present. Therefore, to advance the field, it is necessary to use an objective assay readout in order to properly evaluate and understand the capability of a delivery mechanism [3, 22]. When using toxic cargoes, it is essential to exclude that prepore-to-pore conversion and its toxic effects would lead to an overestimation of the delivery of cargo.

## Conclusions

The toxic effect, which originally hampered a further improvement of the retargeted delivery system, was greatly diminished by a rationally designed new PA variant, PA_wt_-sANTXR-Ac2. Higher total uptake and cytosolic delivery of cargo proteins confirmed the improvement of the system. Exemplarily, we have shown the increase with DARPins as cargo molecules; however, the system can also deliver other proteins which are able to pass through the PA pore. With this improved PA variant, we now aim for a broader range of applications with suitable intracellular drug targets.

## Methods

### Cell lines

Flp-In 293 cells stably overexpressing EpCAM and BirA (Flp-In 293-EpCAM-BirA), RD cells stably overexpressing BirA (RD-BirA), and HT29 cells stably overexpressing BirA (HT29-BirA) were cultured using DMEM. MCF7-BirA and SKBR3-BirA cells were maintained in HAM/DMEM mix (50:50) and RPMI, respectively. All media were supplemented with 10% fetal calf serum and 100 IU/mL penicillin and 100 μg/mL streptomycin. G418 was added to the medium for 3 days after cells were taken in culture, to exclude cells that have lost BirA expression. The following G418 concentrations were used: HT29-BirA, 1000 μg/mL; MCF7-BirA, 400 μg/mL; SKBR3-BirA, 200 μg/mL; and RD-BirA, 200 μg/mL.

### Generation of stable BirA cell lines

The generation of stable cell lines has been described before [10, 19]. RD-BirA cells were generated as a stable pool as described in Verdurmen et al. [19] using 600 μg/mL G418.

### Cloning

Cloning of most constructs used in this study has been described before [10, 19]. LF_N_-eGFP-avi-HA was cloned by amplifying eGFP using primers containing a 5′ SpeI and a 3′ AgeI site for cloning into the SpeI/AgeI-restricted pQIq-LF_N_-avi-HA backbone. PA_m_-sANTXR-Ac2 was generated in the same way as PA_wt_-sANTXR-Ac2 [19].

The construct with a shorter linker between PA_WT_ and sANTXR, termed His_6_-MBP-PA_wt-SL_-sANTXR-Ac2, has been cloned using sequence and ligation-independent cloning (SLIC) [23]. The following primers were used to amplify PCR products of PA_wt_-sANTXR-Ac2 in the linker region: 5′ GCAGG CGAAC GTACC TGGGC AGAAA CCATG GGTCT GAATA CCGCA GATAC 3′ and 5′AGGCT GGGTT TTATG ACCAG 3′ for the first PCR product and 5′ ATTGG TAGCC CTGGT CATAA AACCC AGCCT CGCCG TGCCT TTGAT CTG 3′ and 5′ CTTCC AGCAG TTTCT TACCC AGGTC GGATC CGCTC TGCGC CAGAA TGG 3′ for the second PCR product. The plasmid containing the sequence of PA_wt_-sANTXR-Ac2 was digested using NcoI and BamHI. The linker was shortened from SPGHK TQPGS (G_4_S)_5_ GG to SPGHK TQP.

### Protein expression

The *E. coli* strain BL21 was transformed with the described plasmids for the expression of the constructs. A single clone was picked the next day and used for inoculation of autoinduction medium [24]. The cultures were grown at 25 °C until a stable OD_600_ was reached. Cultures were centrifuged for 10 min at 5000*g* at 4 °C; the pellet washed with PBS, pH 7.4, shock-frozen, and stored at − 20 °C until purification.

### Protein purification

All proteins, expressed as His_6_-MBP-PA variants and His_6_-MBP-LF_N_ cargo constructs, were purified in a similar manner. All steps were performed at 4 °C. Tris-HCl buffers were adjusted to pH 8.0. Bacterial cell pellets were thawed and resuspended in lysis buffer (50 mM Tris-HCl, 0.5 mM EDTA, 0.4 mM 4-(2-aminoethyl)benzolsulfonyl fluoride (AEBSF), 500 mM NaCl, 10 mM MgCl_2_, 1 g/L lysozyme, 10% glycerol, 10 U/mL Pierce™ Universal Nuclease for Cell Lysis) (Thermo Scientific™ 88702). Cells were lysed by sonication and centrifuged for 45 min at 20,000*g*, and the cleared lysate was filtered (pore size 0.22 μm). Proteins were purified by their His-tag via immobilized metal ion affinity chromatography (IMAC). Ni-NTA agarose (Qiagen) was packed in 7 mL benchtop columns (PD10), and columns were equilibrated in lysis buffer, not containing AEBSF and Pierce nuclease. Lysate was applied twice to the column, washed with 10 column volumes (CV) high-salt buffer (25 mM Tris-HCl, 500 mM NaCl, 20 mM imidazole) and 10 CV low-salt buffer (25 mM Tris-HCl, 125 mM NaCl, 20 mM imidazole), and eluted with 2 CV elution buffer (25 mM Tris-HCl, 125 mM NaCl, 300 mM imidazole). Proteins were dialyzed overnight against anion exchange chromatography (AEX) equilibration buffer (25 mM Tris-HCl, 125 mM NaCl) with a 1:10 M ratio of his-tagged Tobacco etch virus (TEV) protease to cleave off His_6_-MBP. TEV protease, MBP, and residual uncleaved proteins were removed via reverse IMAC. For His_6_-MBP-PA_wt-SL_-sANTXR-Ac2, the His_6_-MBP tag was not cleaved off and no reverse IMAC was performed since equivalent delivery to His_6_-MBP cleaved variants of PA_m_-Ac2 has been shown before [19].

The unbound fraction of reverse IMAC was purified via AEX using a MonoQ 5/50 GL (GE Healthcare) on an ÄKTA Pure system (GE Healthcare). Proteins were eluted in a 40 CV gradient up to 50% AEX elution buffer (25 mM Tris-HCl, 1 M NaCl); protein-containing fractions were evaluated by SDS-PAGE, pooled and concentrated via Amicon Ultra-0.5 (Millipore; MWCO 30,000). Subsequently, proteins were polished and buffer exchanged to PBS (pH 7.4) via size exclusion chromatography (SEC) using a Superdex 200 10/300 GL (GE Healthcare). Monomeric fractions were pooled and concentrated as described before. LF_N_-cargo constructs, containing an avi-tag, were additionally incubated with streptavidin beads (Genscript) for 30 min at 4 °C while shaking in order to remove already biotinylated proteins. All proteins were snap-frozen in liquid N_2_ and stored short term at − 20 °C. Purities and monomeric behavior were confirmed to be > 90% by Coomassie-stained SDS-PAGE and on an analytical SEC (Additional file 1: Figure S7).

### Biotin ligase uptake assay

To measure the total cellular uptake and cytosolic localization of cargo proteins, the biotin ligase assay was performed as described previously [22].

### Viability assay

XTT assays were used to evaluate cell viability. Cells were seeded in a flat 96-well plate 48 h before incubation with proteins. Twenty-five thousand cells were seeded for all cell lines. Cells were incubated with respective protein concentrations for 24 h under their normal culture conditions. Cell proliferation was measured with a Cell Proliferation XTT Kit (BIOFROXX) according to the manufacturer’s instructions. Data were plotted as mean ± SEM (*n* = 3).

### Flow cytometry analysis

To determine receptor surface expression levels, cells were incubated on ice in PBS supplemented with 50 mM sodium azide and a 1:50 dilution of the respective antibody for 30 min. Cells were stained with Alexa Fluor 488-labeled anti-EpCAM mouse mAb (VU1D9, Cell Signaling) for EpCAM levels. A reference mouse mAb IgG1 isotype control labeled with Alexa Fluor 488 (MOPC-21, Cell Signaling) was used. EpCAM expression levels were determined on an LSR II Fortessa (BD Biosciences) on gated cells. Data (*n* = 3) were analyzed with GraphPad Prism 8 and plotted as mean ± SEM.

### Confocal microscopy

Forty-eight hours before confocal imaging, 60,000 cells were seeded in 8-well 15 μ-Slide glass bottom slides (Ibidi). PA (100 nM), LF_N_-eGFP (200 nM), and PI (1 μg/mL) were added to the cells with fresh medium. The panel of cell lines was incubated using 50 nM PA. Cells were imaged 3 h after addition of components at 37°C and 5% CO_2_ on a Nikon Eclipse Ti-E inverted microscope with a Yokogawa spinning disc system W1, a 100x oil objective, and an incubation system for live cell imaging including a stage-top incubator for chambered cover glass.

### Time-lapse microscopy

For time-lapse imaging, 25,000 cells were seeded in Nunc MicroWell 96-Well Optical Bottom Plate with Polymer Base (Thermo Scientific) 24 h before imaging. Fifty nanomolar PA, 100 nM LF_N_-eGFP, and PI (1 μg/mL) were added right before imaging to each well (*n* = 6). Cells were imaged at 37 °C and 5% CO_2_ using a LionHeart FX microscope with a 10x objective and the Gen5 software (3.05). Cellular analysis was performed with the Gen5 software in the Texas Red Channel. The threshold for cell detection was set to the image background levels, and object selection was set to 5 μm and 50 μm for min. and max. cell size, respectively. Data were plotted as mean ± SEM (*n* = 6).

## Supplementary information

**Additional file 1: Figure S1.** [Ribbon representation of constructs PA_m_-sANTXR-Ac2 and PA_wt-SL_-sANTXR-Ac2]. **Figure S2.** [Different imaging timepoints of time-lapse imaging experiments]. **Figure S3.** [Figure 2c replotted with additional quantification of LF_N_-eGFP delivery]. **Figure S4.** [Toxicity and delivery of PA_wt-SL_-sANTXR-Ac2]. **Figure S5.** [Gating strategy for receptor expression analysis]. **Figure S6.** [Biotin ligase uptake assay of LF_N_-eGFP]. **Figure S7.** [HPLC analysis of protein purity and monomeric behavior].

**Additional file 2.**

**Additional file 3.** Time-lapse microscopy of Flp-In 293-EpCAM-BirA cells incubated with PAm-sANTXR-Ac2.

**Additional file 4.** Time-lapse microscopy of Flp-In 293-EpCAM-BirA cells incubated with PAm-Ac2.

**Additional file 5.** Time-lapse microscopy of Flp-In 293-EpCAM-BirA cells incubated with PAwt-sANTXR-Ac2.

## Data Availability

The datasets supporting the conclusions of this article are included within the article and its additional files.

## Abbreviations

ADC: Antibody-drug conjugate
DARPin: Designed ankyrin repeat protein
EpCAM: Epithelial cell adhesion molecule
PA: Protective antigen
LF_N_: Lethal factor 1-254
PA_wt_: Wild-type protective antigen
CMG2: Capillary morphogenesis gene-2
TEM8: Tumor endothelial marker-8
VWA: Von Willebrand factor A
PA_m_: Mutated protective antigen (N682A/D683A)
sANTXR: CMG2 receptor VWA domain
PI: Propidium iodide
SL: Short linker
HSP70: Heat shock protein 70
SLIC: Sequence and ligation-independent cloning
AEBSF: 4-(2-Aminoethyl)benzolsulfonyl fluoride
IMAC: Immobilized metal ion affinity chromatography
CV: Column volume
AEX: Anion exchange chromatography
TEV: Tobacco etch virus
SEC: Size exclusion chromatography
