# Supplementary material for: Reengineering anthrax toxin protective antigen for improved receptor-specific protein delivery
Source: BMC Biol. 2020 Aug 13;18:100. doi: 10.1186/s12915-020-00827-y (PMC7427085; doi:10.1186/s12915-020-00827-y)
Supplement: Supplementary file 1 — Additional file 1: Figure S1. [Ribbon representation of constructs PAm-sANTXR-Ac2 and PAwt-SL-sANTXR-Ac2]. Figure S2. [Different imaging timepoints of time-lapse imaging experiments]. Figure S3. [Figure 2c replotted with additional quantification of LFN-eGFP delivery]. Figure S4. [Toxicity and delivery of PAwt-SL-sANTXR-Ac2]. Figure S5. [Gating strategy for receptor expression analysis]. Figure S6. [Biotin ligase uptake assay of LFN-eGFP]. Figure S7. [HPLC analysis of protein purity and monomeric behavior]. [file 12915_2020_827_MOESM1_ESM.pdf]

Additional File 1

Supplementary Figures

for

Reengineering anthrax toxin protective antigen for improved receptor-specific protein delivery

Lukas Becker[1], Wouter P. R. Verdurmen[2], and Andreas Plückthun[1*]

[1]Dept. of Biochemistry, University of Zurich, Winterthurerstr. 190, 8057 Zurich, Switzerland

[2]Dept. of Biochemistry, Radboud Institute for Molecular Life Sciences (RIMLS), Radboud University Medical Center, Geert Grooteplein 28, 6525 GA, Nijmegen, The Netherlands

*Address correspondence to plueckthun@bioc.uzh.ch

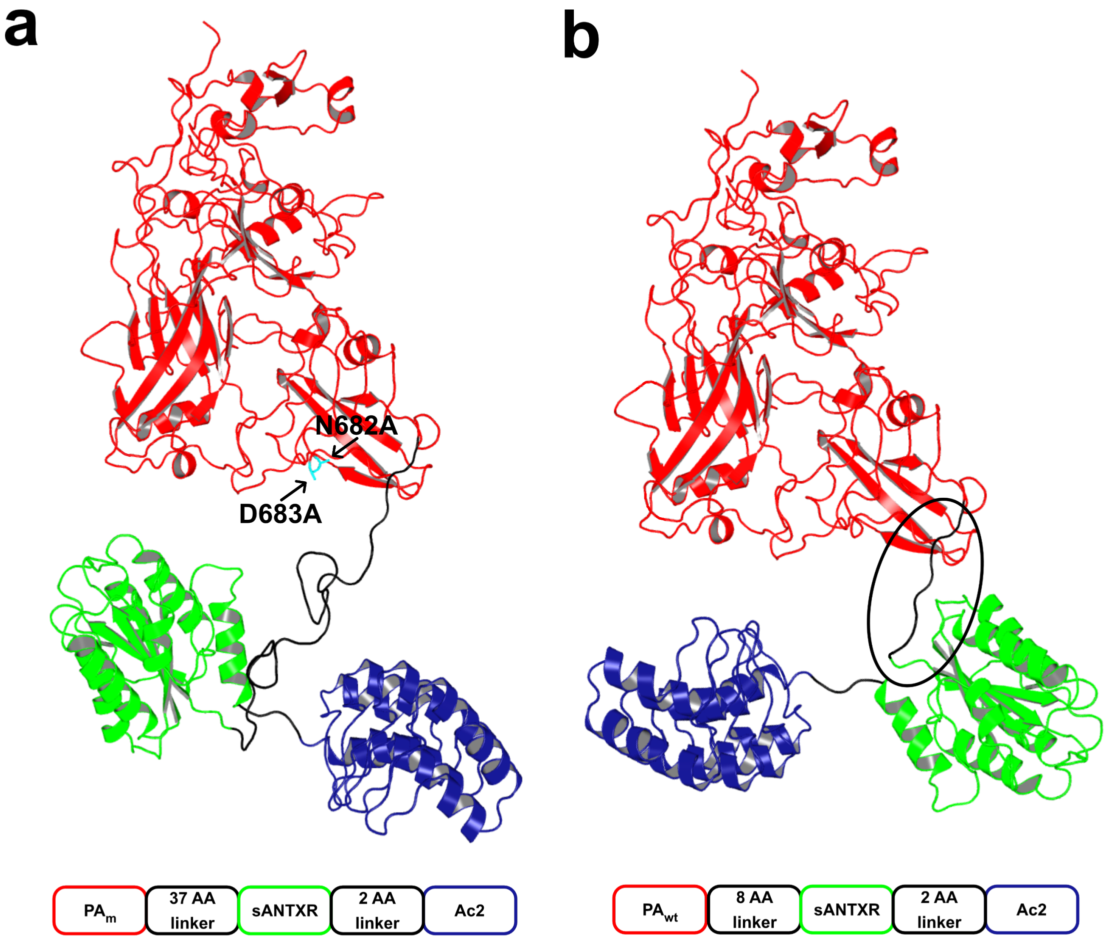

Figure S1: Ribbon representation of the structure of PA$_m$-sANTXR-Ac2 (a) and PA$_{wt\text{-}SL}$-sANTXR-Ac2 (b) shown in their activated/ furin-cleaved PA$_{63}$ version. Either the mutations N682A and D683A (arrows) (a) or the 8 amino acid short linker, highlighted in black oval (b) prevent the interaction of the sANTXR domain with PA. PA is shown in red, the EpCAM-retargeting DARPin Ac2 shown in blue, the sANTXR domain shown in green. Protein structures are adapted from PDB ID: 1TZN (PA prepore binding sANTXR), 1ACC (PA) and 4YDW (DARPin).

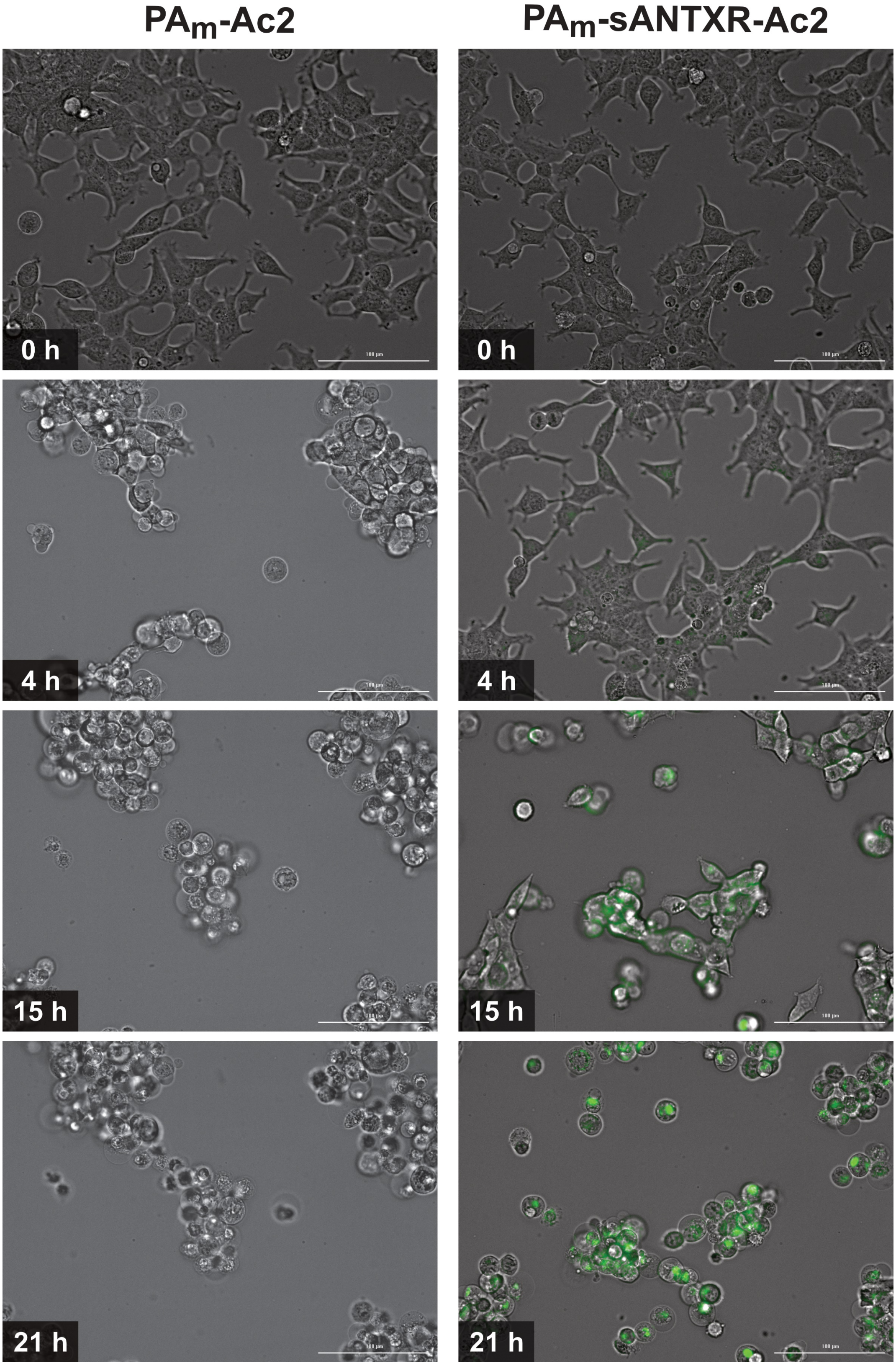

Figure S2: Different timepoints of toxicity testing of different PA-constructs on Flp-In 293-EpCAM-BirA cells stably expressing EpCAM. 25,000 cells were seeded 24 h before imaging. Cells were incubated with 50 nM PA and 100 nM LF-eGFP, which cannot be transported. Cells were imaged using a LionHeart FX microscope with a 20× objective at 37 °C and 5% CO_2.

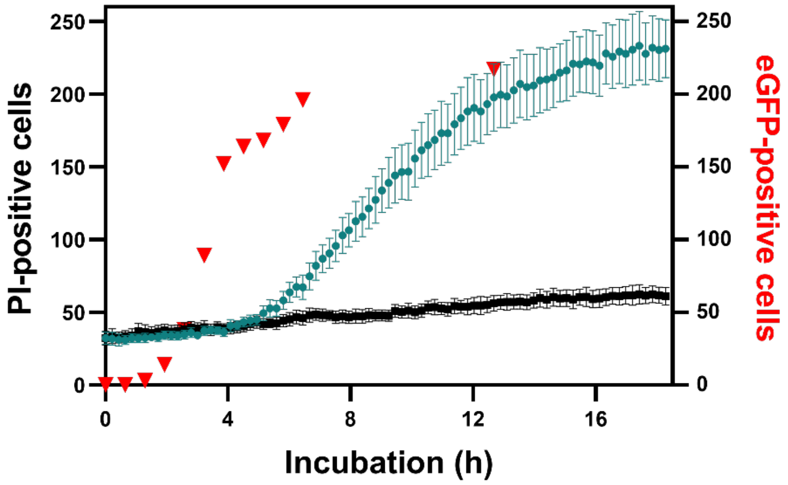

Figure S3: Figure 2c is replotted here with the addition of the quantification (red triangles) of binding of LF_N-eGFP to Flp-In 293-EpCAM-BirA cells during time-lapse imaging. For these measurements, cells treated with PA_{wt}-sANTXR-Ac2 and LF_N-eGFP of a single well staining positive for eGFP were counted at the respective timepoints.

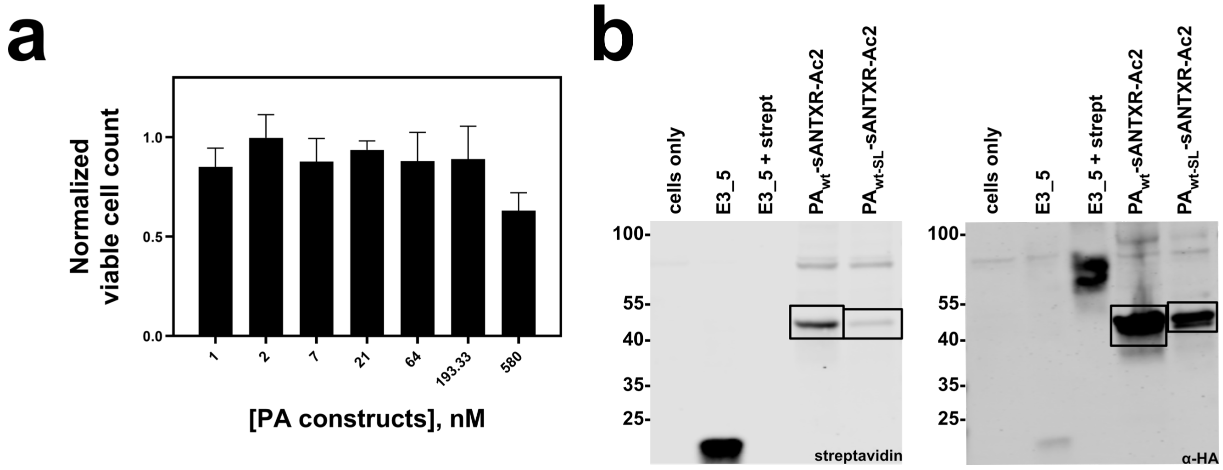

Figure S4: PA$_{wt-SL}$-sANTXR-Ac2, which possibly does not form an intramolecular complex (PA$_7$) efficiently, used as an additional control to PA$_m$-sANTXR-Ac2, tested for toxicity (a) and delivery (b) on Flp-In 293-EpCAM-BirA cells. (a) XTT assay of PA$_{wt-SL}$-sANTXR-Ac2 on Flp-In 293-EpCAM-BirA cells (n=3). Error bars indicate SD; (b) BirA assay of PA$_{wt-SL}$-sANTXR-Ac2 and PA$_{wt}$-sANTXR-Ac2 on Flp-In 293-EpCAM-BirA cells incubated with 100 nM of respective PA and 500 nM of LF-NI$_1$C. Blots show cytosolic cargo, stained with streptavidin IRDye 680LT (left) and total cellular uptake, measured via HA-tag on LF-cargo (right). Lanes labelled as E3_5 *or* E3_5 + strept (streptavidin) *are transiently transfected assay controls for successful biotinylation activity of tested cells.*

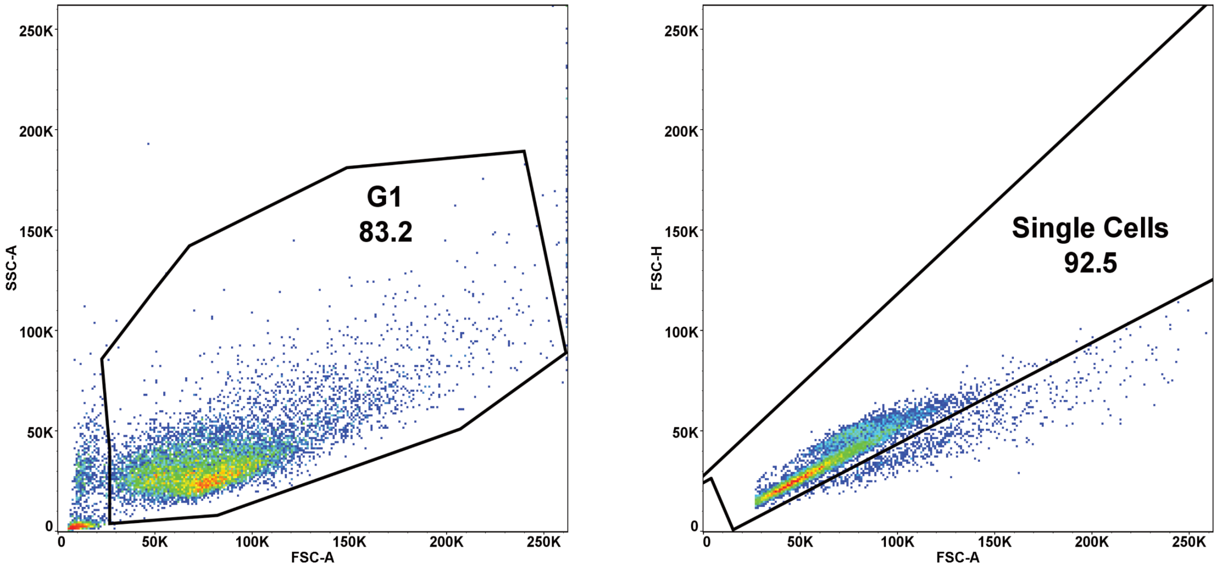

Figure S5: Exemplary flow cytometry gating strategy. Cells expressing EpCAM were incubated on ice in PBS supplemented with 50 mM sodium azide and an Alexa Fluor 488 labelled anti-EpCAM mouse mAb or a reference mouse mAb IgG1 isotype control labelled with Alexa Fluor 488. Flow cytometry analysis was performed and an example of the gating strategy is shown here. The cell population was gated with forward scatter (FSC-A) and side scatter (SSC-A) (gate 1, G1) and single cells were gated via FSC-A and FSC-H (Single cells). Single cells were further analyzed for their EpCAM expression shown in Figure 3.

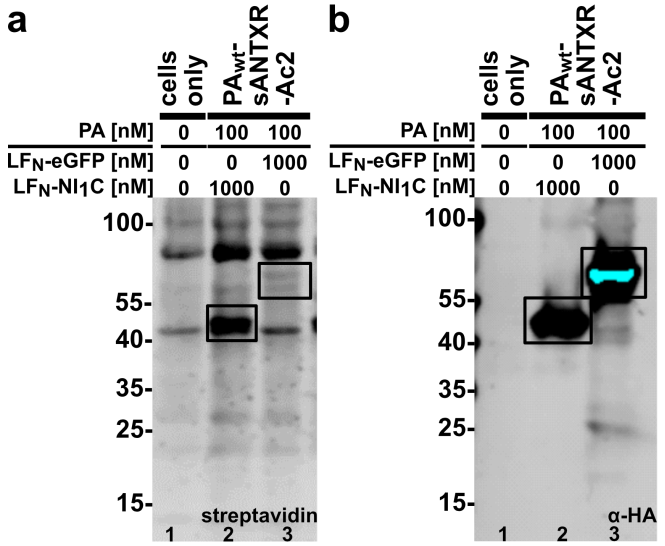

Figure S6: BirA assay of LF_N-eGFP with PA_{wt}-sANTXR-Ac2 on Flp-In 293-EpCAM-BirA cells incubated with 100 nM of PA and 1000 nM of LF_N-eGFP or LF_N-NI_1C, as a positive control for translocation. Blots show cytosolic cargo (a), detected with streptavidin IRDye 680LT and total cellular uptake (b), measured via the HA-tag on LF-cargo.

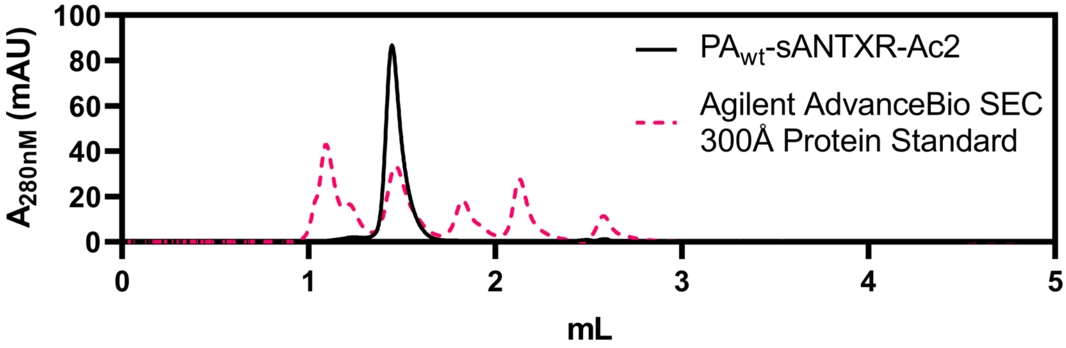

Figure S7: Representative size exclusion HPLC analysis of protein purity and monomeric behavior. Exemplary chromatogram shows PA_{wt}-sANTXR-Ac2 and an Agilent AdvanceBio SEC 300 Å Protein Standard analyzed on a Superdex-200 Increase 5/150GL column.
